# Evaluating the impact of social determinants, conditional cash transfers and primary health care on HIV/AIDS: Study protocol of a retrospective and forecasting approach based on the data integration with a cohort of 100 million Brazilians

**DOI:** 10.1371/journal.pone.0265253

**Published:** 2022-03-22

**Authors:** Davide Rasella, Gabriel Alves de Sampaio Morais, Rodrigo Volmir Anderle, Andréa Ferreira da Silva, Iracema Lua, Ronaldo Coelho, Felipe Alves Rubio, Laio Magno, Daiane Machado, Julia Pescarini, Luis Eugênio Souza, James Macinko, Inês Dourado

**Affiliations:** 1 Institute of Collective Health, Federal University of Bahia, Salvador, Brazil; 2 ISGlobal, Hospital Clínic - Universitat de Barcelona, Barcelona, Spain; 3 Center for Data and Knowledge Integration for Health (CIDACS), Gonçalo Moniz Institute, Oswaldo Cruz Foundation (FIOCRUZ), Salvador, Brazil; 4 Department of Chronic Conditions and Sexually Transmitted Infections/Department of Health Surveillance/Ministry of Health (DCCI/SVS/MS), Brasília, Brazil; 5 Life Science Department, University of the State of Bahia, Salvador, Brazil; 6 UCLA Fielding School of Public Health, University of California at Los Angeles (UCLA), Los Angeles, California, United States of America; Public Library of Science, UNITED KINGDOM

## Abstract

**Background:**

Despite the great progress made over the last decades, stronger structural interventions are needed to end the HIV/AIDS pandemic in Low and Middle-Income Countries (LMIC). Brazil is one of the largest and data-richest LMIC, with rapidly changing socioeconomic characteristics and an important HIV/AIDS burden. Over the last two decades Brazil has also implemented the world’s largest Conditional Cash Transfer programs, the Bolsa Familia Program (BFP), and one of the most consolidated Primary Health Care (PHC) interventions, the Family Health Strategy (FHS).

**Objective:**

We will evaluate the effects of socioeconomic determinants, BFP exposure and FHS coverage on HIV/AIDS incidence, treatment adherence, hospitalizations, case fatality, and mortality using unprecedently large aggregate and individual-level longitudinal data. Moreover, we will integrate the retrospective datasets and estimated parameters with comprehensive forecasting models to project HIV/AIDS incidence, prevalence and mortality scenarios up to 2030 according to future socioeconomic conditions and alternative policy implementations.

**Methods and analysis:**

We will combine individual-level data from all national HIV/AIDS registries with large-scale databases, including the “100 Million Brazilian Cohort”, over a 19-year period (2000–2018). Several approaches will be used for the retrospective quasi-experimental impact evaluations, such as Regression Discontinuity Design (RDD), Random Administrative Delays (RAD) and Propensity Score Matching (PSM), combined with multivariable Poisson regressions for cohort analyses. Moreover, we will explore in depth lagged and long-term effects of changes in living conditions and in exposures to BFP and FHS. We will also investigate the effects of the interventions in a wide range of subpopulations. Finally, we will integrate such retrospective analyses with microsimulation, compartmental and agent-based models to forecast future HIV/AIDS scenarios.

**Conclusion:**

The unprecedented datasets, analyzed through state-of-the-art quasi-experimental methods and innovative mathematical models will provide essential evidences to the understanding and control of HIV/AIDS epidemic in LMICs such as Brazil.

## Introduction

It is estimated that 37.7 million people are currently living with the HIV/AIDS and 680 thousand die annually worldwide [1]. While, after 40 years form its appearance, the large number of effective treatments and prevention interventions have implemented, causing a considerable reduction in new cases in the world, this reduction did not occur equally among populations, subpopulations and geographic regions [2]. In order to reduce these inequalities, and achieve the AIDS-related Sustainable Development Goal (“end the epidemic of AIDS by 2030”), structural interventions should aim to reduce poverty, social vulnerabilities, and decrease geographic and economic barriers to healthcare [3]. Recent studies suggest that Conditional Cash Transfers (CCTs) can be effective for the reduction of maternal transmission of HIV, may reduce HIV/AIDS incidence among some populations, and can increase adherence to ARV treatment [4–6]. Such programs may benefit those with HIV/AIDS through improved nutritional status and living conditions, and by reducing barriers to access education and healthcare services—but results are far from conclusive. To date existing studies do not all show significant or even positive impacts, mostly have weak generalizability due to small study samples and the specific populations under study—mainly in areas of Sub-Saharan Africa [7–9]—and, due to the need for large and long-lasting cohorts, there are no current studies evaluating the impact of CCTs on HIV/AIDS-related hospitalizations and mortality. At the international level, while having a usual source of primary health care (PHC) has been associated with numerous beneficial health outcomes, the majority of the current literature focuses on primary health care as either a place to provide voluntary counselling and testing and/or as a potential location for provision of antiretroviral medications [10]. Despite recent calls for strengthening primary health care as the basis for universal health coverage, there is little evidence of how individuals with HIV/AIDS may benefit from strong, community-based primary health care services (both for HIV/AIDS care as well as for a myriad of other health needs) [11]. There is no evidence whatsoever regarding the potentially synergistic effects between CCTs and PHC on HIV/AIDS incidence, prevalence, hospitalizations and mortality even though both approaches are becoming increasingly common in middle-income and some lower-income countries [12, 13].

Brazil is one of the largest and most inequal Low and Middle-Income countries, it implemented during the last decades a number of well-evaluated programs intended to meet the needs of people living with HIV/AIDS, and its comprehensive approach has been regarded as relatively successful [14, 15]. Nevertheless, the HIV/AIDS epidemic is still burdensome, with a total of 835.791 AIDS cases notified in 2020, 50% of them distributed among the overall heterosexual population and other 50% concentrated in vulnerable groups such as men who have sex with men (MSM), drug users (DUs), and commercial sex workers. While the country has made considerable progress in addressing the improvement of social determinants of health and reducing poverty, since 2014 a sharp and deep recession in Brazil has unfolded, with annual GDP contractions of 3.8% and 3.6% in 2015 and 2016, respectively [16]. The economic crisis led to increasing unemployment, mainly among low-income populations, and a severe inversion of the previous trends of poverty and inequality reduction. This context provides the opportunity to explore the role of social protection programs in maintaining the health and the wellbeing of vulnerable populations living with HIV/AIDS during periods of economic crises.

The Brazilian conditional cash transfer (CCT) program, Bolsa Familia Program (BFP), is the world`s largest CCT and targets poor households earning between US$18–36 per person per month. Eligibility is assessed at the federal level to minimize errors and fraud. The monthly cash benefits range from US$17 to a maximum of US$41 (depending on household size and composition) and payments are credited directly to a beneficiary debit card [17, 18]. Conditions for receipt of the benefits include: attendance at prenatal and postnatal monitoring sessions; children aged 0–7 must be up-to-date with nutrition monitoring and immunizations, and children must attend school. Previous studies have assessed the effectiveness of the BFP on child morbidity and mortality and a host of other conditions, from suicide and tuberculosis to leprosy [19–23]. Brazil has also implemented over the last two decades one of the most consolidated and evaluated Primary Health Care (PHC) programs, the Family Health Strategy (FHS), in a comprehensive approach to providing PHC as part of Brazil’s National Health Services, the Unified Health System (SUS) [24]. These facilities are open to all Brazilian citizens and are located within community settings close to where people live. Several studies have evaluated FHS effectiveness, demonstrating that expansion of the FHS has contributed to better health outcomes, including reducing infant and adult mortality, decreasing unnecessary hospitalizations, enhancing health system functions such as recording of vital statistics, and decreased inequities in both healthcare access and health outcomes [25–32].

Brazil is also an extremely data-rich country, where demographic, socioeconomic and interventions coverage data are available in terms of both quantity and adequate quality. Several impact evaluations of CCT have been performed using publicly available secondary aggregate-level longitudinal data and, more recently, individual-level data using the 100 Million Brazilian Cohort [33]. For these reasons Brazil provides a unique opportunity to study the effects of changes in socioeconomic determinants, CCT and PHC on a such complex and chronic infectious disease as HIV/AIDS in LMIC. Therefore, the detailed objectives of the proposed study are described as follows:

**Aim 1**
  To integrate aggregate and individual-level large datasets to evaluate the effect of a broad range of socioeconomic determinants on incidence, treatment adherence, hospitalization, case fatality and mortality rates from HIV/AIDS;
**Aim 2**
  To assess the impact of BFP exposure and FHS coverage on incidence, treatment adherence, hospitalizations, case-fatality, and mortality from HIV/AIDS, evaluating their individual and synergistic effects on a broad range of subpopulations;
**Aim 3**
  To forecast HIV/AIDS incidence, prevalence and mortality scenarios in Brazil using integrated microsimulation (MS), compartmental model (CM) and agent-based models (ABM) that leverages the large set of results from Aims 1 and 2, to explore the impact of different socioeconomic and policy scenarios on HIV/AIDS morbidity and mortality up to 2030.

### Innovation of the study

This study breaks new ground by providing a unique opportunity to use unprecedented big longitudinal dataset of linked socioeconomic and health data to assess the effects of socioeconomic conditions, PHC and CCT on all sequential outcomes of HIV/AIDS morbidity and mortality. Moreover, this study provides the unique opportunity to assess potential effect modification and synergistic effects of the simultaneous receipt of CCT and PHC, and how baseline socioeconomic conditions influence the effectiveness of both types of programs on the burden of HIV/AIDS. The longitudinal aspect of the cohort, which has a long follow-up period of 18 years of socioeconomic and health data, will allow us to explore and assess in-depth dynamics, lagged and long-term effects of changes in living conditions and in exposures to BFP and PHC. One of the study’s most relevant potential contribution to translational science will be the systematic integration of the large dataset and results from the retrospective impact evaluations with the prospective mathematical models, developing more reliable forecast scenarios than models which use parameters from other studies or populations. Moreover, the creation of a pioneering hybrid microsimulation, compartment and agent-based model (MS-CM-ABM), which will be supported by the very large number of parameters available from the retrospective big data evaluation, will be an innovation in the epidemiology of infectious diseases, and will be essential to the effort of modelling the effects of social determinants, CCT and PHC on HIV/AIDS. To date, very few studies in HIV/AIDS have introduced poverty and CCT as variables in their predictive models, and they have not explored their influence in depth [34, 35]. Regarding PHC, no models have assessed the impact of access to universal PHC on HIV/AIDS-relevant outcomes (beyond universal HIV screening in primary care settings) [36], neither have mathematical models been developed to estimate these effects.

## Materials and methods

### Conceptual framework

**Fig 1:**
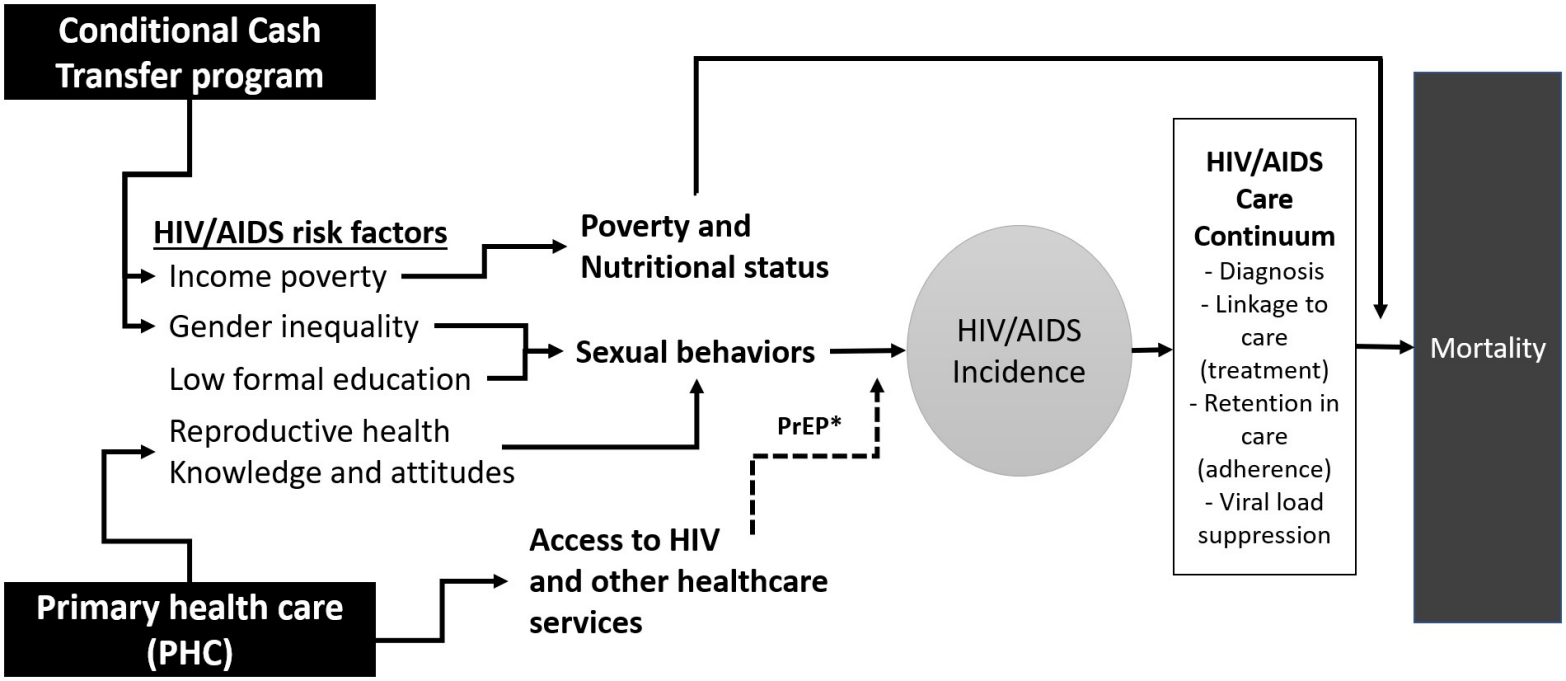
Conceptual framework of selected structural determinants of HIV/AIDS incidence and mortality, and of the hypothesized effects of cash transfer and PHC on this process. *Note: Pre-Exposure Prophalaxis (PrEP) is not yet widespread in Brazil and cannot yet be assessed in this study.

The conceptual framework for the project is presented in Fig 1. HIV transmission is linked with high-risk sexual behaviours that are associated with structural social determinants of health including income, poverty, gender inequality, and low education [4–6]. Interest in CCT grew for HIV/AIDS from its potential to mitigate income poverty, increase children’s school attendance, and promote health-seeking behaviours. The role of health services in HIV care has been most widely discussed within the context of the HIV care continuum, which requires accessible healthcare services providing voluntary counselling and testing, linking individuals to care, receipt of antiretrovirals and other essential elements of HIV care, and adherence to ARTs, all of which should lead to viral suppression.

In the case of Brazil, the FHS could complement HIV clinic-based providers of HIV prevention and care services by increasing the proportion of its patients who have been screened for HIV, opening dialogues around sexual health, including asking about sexual orientation and gender identity, and prescribing antivirals as pre- and post-exposure prophylaxis for their non-HIV-infected patients [37]. PHC could also reduce HIV/AIDS mortality through earlier detection and treatment of the disease, enhancing adherence to treatment, and facilitating earlier detection and treatment of complications [38, 39].

### Data and measures

**Fig 2:**
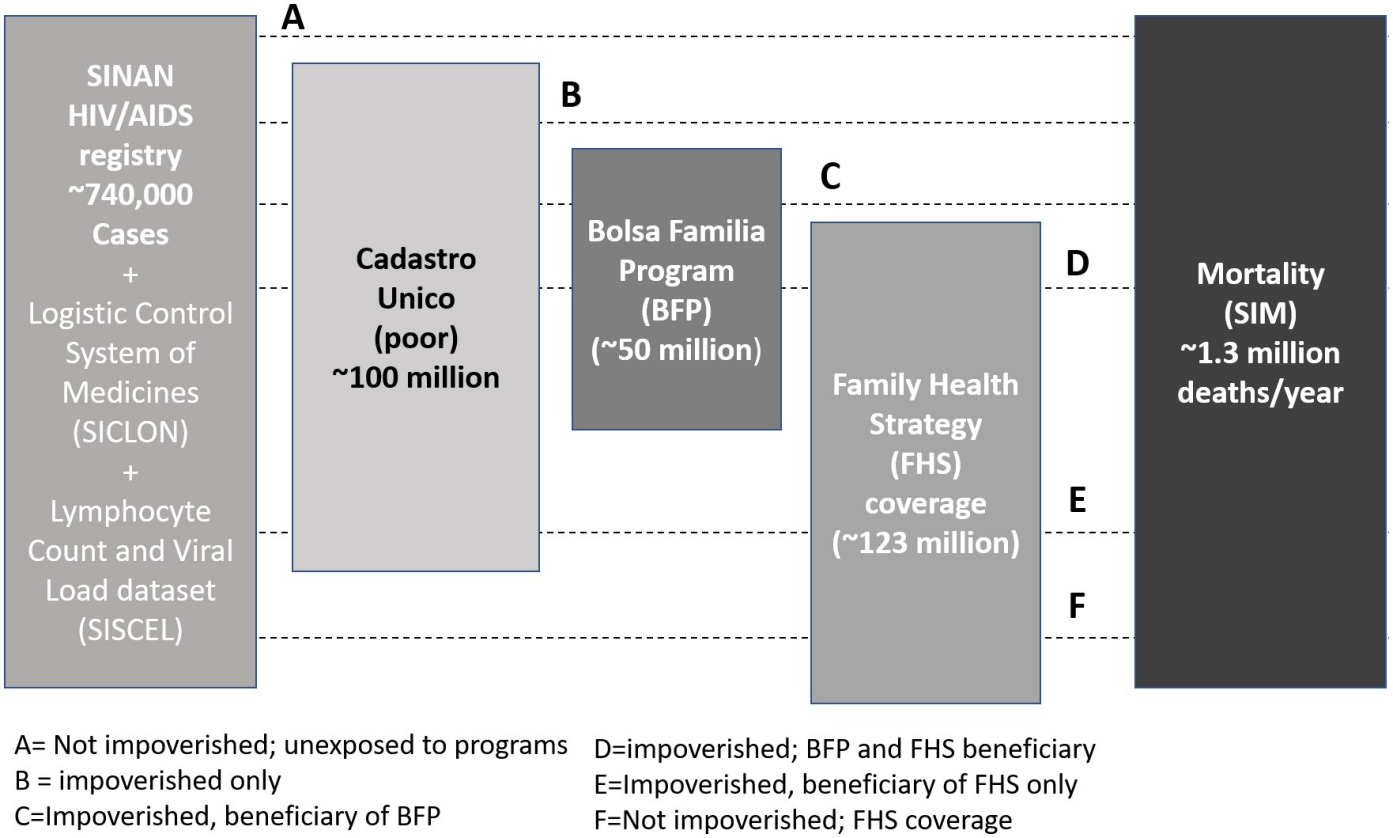
Datasets for linkage and subsequent study cohorts.

This study builds upon the integration of a wide range of datasets. The aggregate-level longitudinal data on municipalities conditions, infrastructures, healthcare coverage and morbidity and mortality levels will be integrated with large individual-level dataset constituted by the linkage between AIDS-specific clinical and treatment longitudinal data with the 100 Million Brazilian Cohort, built by the Centre for Data and Knowledge Integration for Health (CIDACS), Salvador, Brazil [40, 41]. Fig 2 illustrates the different datasets linked and the different population groups that will be compared. The cohort has information of over 100 million individuals that were registered during 2000 and 2018 in *Cadastro Unico* (*CadUnico*), a national administrative system containing information on all individuals, and their families, applying for any social programs in Brazil [42]. To be eligible for registration in CadUnico, families must earn a per capita income of up to half the minimum wage or a total familial income of up to 3 times the minimum wage (e.g., minimum wage ranged from 380 BRL in 2007 to 724 BRL in 2014). By the end of 2018, the CadUnico contained approximately 114 million individual registrations, representing just over 50% of the Brazilian population. At registration, enrollees are assigned a unique numerical identifier and surveyed regarding socioeconomic indicators. The CadUnico collects information about housing conditions, income and demographic characteristics of all the members of a family registered. The CadUnico cohort includes 246 variables with information from the family and individuals registered in this data collection and storage system [43]. The data include socioeconomic aspects such as income, occupation, and employment status; demographic characteristics such as age, schooling, ethnicity, marital status, family composition, and number of families in the same household; living conditions such as number of rooms in the house, housing material, household water supply, sewage disposal system, electricity in the home, and waste collection; as well as information regarding health status as presence of a mental health disorder, disabilities and access to healthcare services. The CadUnico also holds information about who has received the BFP benefit, when it was received, and the total amount received per month.

The 100 Million Brazilian Cohort is based on the linkage between the *CadUnico* with the Brazilian Mortality Information System (SIM), that provides all death notifications by age, sex, cause (including AIDS), and municipality of residence of the deceased. More recently, the *CadUnico* was also linked with the Brazilian Hospitalization Information System (SIH), that provides registers for all, public hospitalizations, including for AIDS.

The health datasets related to HIV/AIDS and that will be linked to 100 Million Brazilian Cohort and be used in this study are described below.

1. The National System of Disease Notification for AIDS (SINAN), that includes information on all notified AIDS cases in the country.
2. The Logistic Control System of Medicines (SICLOM), that manages the logistics of all antiretroviral drugs in Brazil and report when and how many doses are provided to the patients.
3. The National Network System of Laboratories of CD4 + CD8 + Lymphocyte Count and Viral Load (SISCEL), that records all the CD4/CD8 counts and viral load among people living with HIV and AIDS.

The CIDACS has developed algorithms and codes to perform efficient, highly sensitive and specific probabilistic linkages using each individual’s name, date of birth, sex, and municipality of residence between these systems, and has successfully tested linkages [44, 45]. The quality of the each linkage between *CadUnico* and SIM (for all causes) have been extensively evaluated and validated [46, 47]. SINAN-AIDS, SICLOM and SISCEL, which have not been previously linked to the 100 Million Cohort because only HIV/AIDS specific, will be linked using the same algorithms and the linkages will be validated using the same protocols. This study will be conducted in accordance with Resolution N° 466/2012 of the National Health Council (CNS), and it was approved by the Research Ethics Committee of the Institute of Collective Health (ISC) of the *Universidade Federal da Bahia* (UFBA), Salvador, Brazil (Number 41691315.0.0000.5030; Opinion n°: 3.783.920; Date of approval: 21^th^ January 2020).

### Analytic strategy

To achieve the three main objectives of this study, several retrospective and forecasting methods will be integrated. The detailed steps of each objective are described below. As discussed before, the Aim 1 is to use very large datasets to evaluate the effect of a broad range of socioeconomic determinants on incidence, treatment adherence, hospitalization, case fatality and mortality from HIV/AIDS. A hierarchical framework to investigate the social determinants of HIV/AIDS [40, 48] will be developed. Social indicators will be grouped into three blocks representing distal, intermediate, and proximal variables. Distal variables are related to geographical factors and include region and location of the family household (urban or rural). Intermediate variables are related to socioeconomic position in the community and include self-identified race or ethnicity, education, employment, and *per capita* family income. Proximal variables are related to household conditions at the family level and include building material, water supply, sanitation, electricity, waste disposal destination, and household density. Following a hierarchical analytical approach, three adjusted Poisson regression models will be used with cluster-robust standard errors (SEs) [49–51], i.e., accounting for familial clustering of covariates, to estimate the adjusted incidence rate ratios (IRRs) of morbidity or mortality HIV/AIDS outcomes. All variables associated with Incidence Rate Ratio of the outcomes at a significance threshold of p-value less than 0.10 will be included in the next level model. Model 1 will include distal factors plus age and sex. Model 2 will include variables from model 1 and intermediated factors. Model 3 will include variables from model 2 and proximal factors.

The second aim of this study is to assess the impact of the Bolsa Familia Program exposure and Primary Health Care coverage on incidence, treatment adherence, hospitalization, case fatality, and mortality from HIV/AIDS, evaluating their impact, alone and in a synergistic fashion, on a broad range of subpopulations. To achieve this goal, the following steps will be performed:

**Step 1**. To analyze the differential impact and dose-response of BFP according to the duration of the intervention, amount of money received and compliance with conditionalities. The retrospective impact evaluation will use arguably one of the most robust study designs for causal inference in quasi-experimental studies: the regression discontinuity design (RDD), which allows a quasi-randomization of the subjects under study around the eligibility threshold [52, 53]. The eligibility of the BFP is determined by two thresholds: one for poor families (below 154 BRL *per capita*) with vulnerable members (children or pregnant women), the second for extremely poor families (below 77 BRL *per capita*), even without any vulnerable member. BFP is one of the most accurately targeted CCTs, and if inclusion/exclusion errors are identified, a fuzzy-instead of sharp-RDD will be used. The presence of two different cut-offs will allow to evaluate the different impact of the BFP on the two different thresholds: between eligible and non-eligible poor and eligible and non-eligible extremely poor families. In order to explore the impact in subjects outside of the RDD selected bandwidth, analyses using Random Administrative Delays (RAD) and Propensity Score Matching (PSM) [54–56] will also performed. These methods have been already used for similar studies with the CadUnico to evaluate the effects of BFP on Tuberculosis (TB). The PSM has also been used in with the same linked dataset to complementarily evaluate the impact of BFP on TB cure and mortality rate [21]. Where possible, combination of these designs—such as the PSM-RDD—will be used [57]. The complementary use of the three methods will consequently allow to assess the effects of BFP along the complete range of socioeconomic conditions represented over the 100 million individuals of the dataset. All analyses above will be stratified according to the years of exposure to BFP and amount of received money over the 12 years of follow-up, to evaluate a dose-response relation with the duration and intensity of support received. Analyses will also explore lagged effects by associating exposures of previous years with outcomes of a range of following years.
**Step 2**. To measure the BFP impact on a broad and detailed range of different subpopulations defined by individual demographic and socioeconomic characteristics and by local geographic regions, and municipality development. The evaluation designs and methods described above will be used for stratified analyses with each subpopulation of interest: age groups, gender, illiteracy and educational level. Further stratifications will be based on the characteristics of the municipality: human development index, GDP per capita and Gini index of income inequality.
**Step 3**. To analyze the dose-response of FHS according to the extent and duration of the municipal coverage. Using the same structured dataset from the previous analyses of Aim 2, and considering that coverage of FHS is only available at the municipal level, the FHS impact will be evaluated using a Propensity Score (PS) weighted multilevel negative binomial regression design with individual level-variables from Aim 1, including BFP as adjusting variable [56]. To avoid ecologic fallacy, the dataset will be resized and limited to municipalities with 90–100% (consolidated FHS coverage = exposed) and 0–10% FHS coverage (low/insignificant FHS coverage = control) in the study period. The intervals 0–10% and 90–100%, instead of 0% and 100%, will be used to consider uncertainty in the coverage measure, but sensitivity analyses will be performed with 0% and 100%.
**Step 4**. To measure the FHS impact on a broad range of different subpopulations defined by individual demographic and socioeconomic characteristics and by local geographic regions. As in the Step 2 of the Aim 2, the evaluation designs and methods will be used for each subpopulations of interest: age groups, gender, illiteracy and educational level. Moreover, further stratifications also will be based on the characteristics of the municipality.
**Step 5**. To evaluate the synergistic individual-level exposure to BFP with municipal-level coverage of FHS on incidence and mortality from HIV/AIDS. Using the same multilevel and multivariable regression models above, the interaction effects of BFP with FHS will be evaluated with product terms and stratification analyses.

The main limitation of Aim 1 and Aim 2 is the usage of the population registered in the CadUnico that will be used rather than the overall Brazilian population to evaluate the impact of the interventions under study. While for the evaluation of the BFP the use of a control group which is part of the poorest half of the Brazilian population—instead of the overall population—is supposed not to influence the impact estimates of RDD and PSM designs (which uses controls comparable to the exposed), for FHS this is not necessarily the case. While the use of PS weighted regression will be able to weight observations and allow an unbiased estimation of the FHS effects (in relation to implementation bias), we assume that the FHS effect in the upper income group of the CadUnico—which includes a great part of the Brazilian middle-class—will not be significantly different from the richest half of the Brazilian population not included in the CadUnico dataset. All analyses for Aims 1 and 2 will be performed with STATA 16^®^ and R Studio 3.6^®^.

Finally, to achieve Aim 3, some steps are needed to be developed. This objective is to forecast HIV/AIDS incidence, prevalence and mortality scenarios, using the broad range of subpopulations estimates and parameters from the retrospective analyses, according to socioeconomic changes and coverage intervention scenarios up to 2030. Leveraging the richness and granularity of parameters obtained from Aim 1 and 2, mathematical models will fully explore and comprehensively evaluate the effect of several scenarios using compartmental models, microsimulation, and agent-based models. The steps are described below.

**Fig 3:**
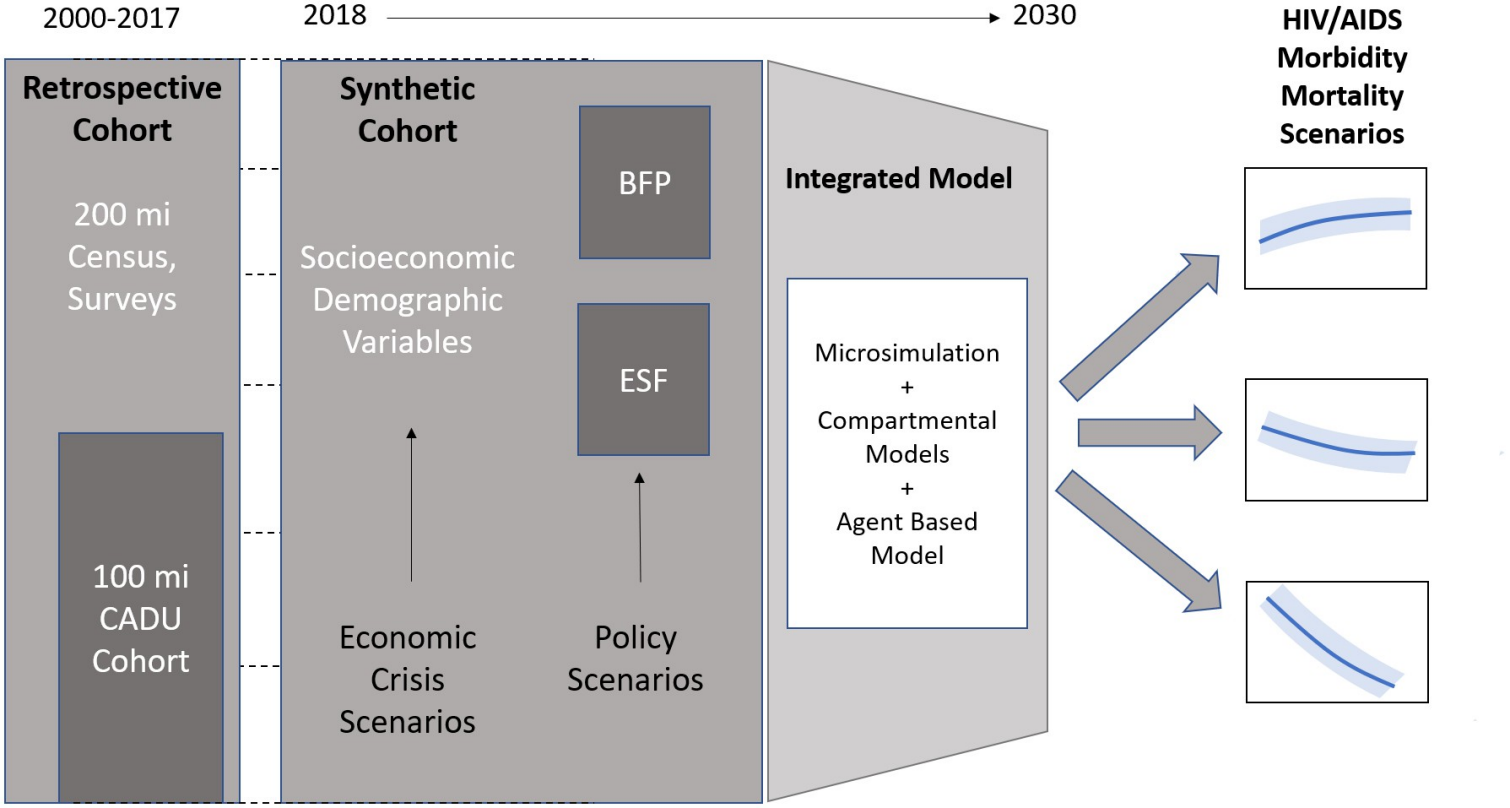
Integrated microsimulation, compartmental and agent-based model.

**Step 1**. To evaluate the most adequate compartmental structure for the mathematical models. In the first exploratory stage we will use compartmental models (CM) to evaluate which structures and compartments are more adequate to represent the influence of the wide range of social determinants, the effects of BFP and FHS on incidence, prevalence, compliance to treatment in the HIV/AIDS epidemic in Brazil as a whole.
**Step 2**. To develop an Agent-Based Model (ABM) to evaluate the effect of interactions in HIV/AIDS dynamics. ABM will be used to explore patterns of interactions between individuals [58]. ABM focuses on understanding population dynamics, and models individuals who “choose” actions under the conditions and environment where they are situated. ABM are increasingly used in public health [59–61] and in HIV/AIDS studies [62–65].
**Step 3**. Develop an integrated Microsimulation, Compartmental and Agent-Based Model (MS-CM-ABM) and forecast the impact of alternative economic and policy scenarios on HIV/AIDS outcomes. The third stage of modelling of the Aim 3 will integrate the two analyses above with microsimulation (MS), creating an integrated and comprehensive model. MS-CM-ABM can provide more accurate estimates of policy effects than traditional compartmental models or time-series forecasting models, which use only the average values in the population. Both MS and ABM use the micro-level description to represent the real phenomena, but while ABM is focused on the understanding the interaction dynamics in relatively small population, MS is used to recreate large synthetic populations from retrospective datasets. Their integration can be particularly useful—as in our case—when both the heterogeneity and dynamic interactions of the population are relevant for the outcome under study. A representation of the proposed MS-CM-ABM model is presented in Fig 3. Despite the growing interest in the integration of the two approaches, few studies have employed this novel approach [66–68].

For each outcome and each scenario, 10,000 Monte Carlo simulations will be performed allowing all parameters to vary by their underlying distribution—measured in the retrospective analysis—and consequently obtaining the Predictive Intervals of each estimate. The linear regression of predicted versus observed values and the proportion of variance (R^2^) explained will be assessed, and will be verified that all observed values will be included in the 95% CIs of the simulation estimates, among other validation criteria [69, 70].

Once the MS-CM-ABM models are validated, we will use them to:

1. Forecast the prolonged effects of the ongoing increases in poverty rates due to the economic crisis and the impact of reductions in BFP and FHS coverages due to the currently implemented fiscal austerity measures, versus different dynamics of improved socioeconomic scenarios and stabilization or increases in BFP and FHS coverage up to 2030.
2. Evaluate in which specific subpopulation groups increased BFP and FHS coverage could be more beneficial. Using the granularity of MS-ABM and subpopulation specific effect estimates from the retrospective evaluation several scenarios of expansion of BFP and FHS in target populations will be tested, and the benefits compared.
3. Evaluate with comprehensive calibration which combination of socioeconomic determinants changes and BFP and FHS subpopulations coverage will allow to reach AIDS-related Sustainable Development Goal 3.3 in Brazil. Using the comprehensive set of scenarios evaluated above, the combinations of socioeconomic changes and interventions expansions will be more finely tuned to maximize the chances to reach the end of HIV/AIDS epidemic in Brazil.

Scenarios will be compared along ratios of HIV/AIDS incidence, prevalence and mortality, and the number of averted cases and deaths. The main potential limitation of our comprehensive MS-CM-ABM models is that they are built upon effect estimates coming from the poorest half of the Brazilian population, while our models will forecast the entire HIV/AIDS burden of the country. As mentioned above, the inputs for modelling will be integrated with parameters from nationally representative studies and there are solid reasons why we assume FHS and BFP effects obtained in Aim 1 and Aim 2 represent the impact on the overall Brazilian population. We will use R Studio^®^ 3.6 and JAS-mine^®^ [71] for MS, CM and ABM and their integration in Aim 3.

## Conclusion

This comprehensive study will provide an innovative approach to assess the effects of several large and successfully scaled programs on HIV/AIDS: the use of unprecedented longitudinal big datasets and the pioneer integration between retrospective and forecasting evaluations, together with innovative hybrid microsimulation, compartmental and agent-based models, will represent an advance in the understanding of HIV/AIDS epidemic, and will provide robust evidences for the implementation of control measures to achieve the AIDS-related SDGs in LMICs.

## Supporting information

S1 Checklist (PDF)
